# The consumption of dietary supplements in Saudi Arabia during the COVID-19 pandemic: A cross-sectional study

**DOI:** 10.1016/j.jsps.2023.101779

**Published:** 2023-09-09

**Authors:** Wedad Azhar, Kholod Al-Otaibi, Wafaa F. Abusudah, Firas Azzeh, Alaa Qhadi, Walaa E. Alhassani, Najlaa H. Almohmadi, Taqwa Bushnaq, Bayan Tashkandi, Nouf Abdullah Alharbi, Abrar Babteen, Mai Ghabashi, Yara Kamfar, Khloud Ghafouri

**Affiliations:** a Clinical Nutrition Department, Faculty of Applied Medical Sciences, Umm Al-Qura University, Makkah, Saudi Arabia; b Department of Food Science and Nutrition, College of Sciences, Taif University, Taif, Saudi Arabia; c Food and Nutrition Department, King Abdulaziz University, Jeddah 22254, Saudi Arabia; d Department of Nutrition and Food Sciences, Northern Border University, P.O. Box 1321, Arar 91431, Saudi Arabia; e Pharamcy Department, King Abdullah Medical City, P.O. Box 57657, Makkah 21955, Saudi Arabia

**Keywords:** Supplements, Quarantine, Immunity, Home confinement, COVID-19, Minerals, Saudi Arabi

## Abstract

**Background and Objectives:**

People frequently utilize dietary supplements (DS), notably during the COVID-19 epidemic, despite the lack of data supporting their usefulness and safety in enhancing general health. This study aimed to measure the consumption of DS in Saudi Arabia during COVID-19. Previous studies focused on using dietary supplements for preventing or reducing mental health.

**Materials and Methods:**

A cross-sectional study was conducted with 1572 participants aged 18–65, from all-over Saudi Arabia who were invited to complete a self-administered questionnaire to measure the consumption of supplements during the pandemic lockdown period. Also, it investigates the factor that effect supplements consumption.

**Results:**

Around 63% of the participants did not change their consumption patterns during the pandemic, while 16% consumed more and 21.4% consumed fewer supplements during the pandemic. The most commonly used supplements were iron; calcium, especially among pregnant women; omega-3, especially in people older than 65 years; and vitamin D. Females (81%; *P* < 0.002); age (94.7%; *P* < 0.002); married people (84%; *P* < 0.001); those with a higher educational level (83.9%; *P* = 0.02); those with a higher monthly income (86.1%; *P* = 0.006); and pregnant women (100%; *P* < 0.007) reported the highest rates of consumption.

**Conclusion:**

Dietary supplements have recently become popular in Saudi Arabia, but large differences remain between Saudis in their consumption of supplements. Additional research should be conducted to examine the level of knowledge of dietary supplements among Saudi population.

## 1 Introduction

According to the Dietary Supplement Health and Education Act (DSHEA), dietary supplements are defined as any products that contain one or more nutritional ingredients that help add nutritional value and supplement the diet (Rehnquist, 2003). In additional to sources of nutrients they have physiological effect that are marketed in “dose” form (e.g. pills, tablets, capsules, liquids). In addition to vitamins and minerals, dietary supplements can also include other less recognizable substances – herbs, amino acids, and enzymes – that improve nutrient levels (Rehnquist, 2003).

The use of dietary supplements has been rising yearly. In Japan, only 32% of adults were consuming dietary supplements (Kobayashi et al., 2017). In India, around 68.3% of the participants consumed multivitamin supplements (Sekhri and Kaur, 2014). Similarly, the use of dietary supplements in the Gulf region is growing over time (Altamimi, 2019), and consumers are taking an interest in the matter (O’Shea and Bindman, 2016).

Saudi Arabia's food supplement market is expanding, perhaps due to a rising population and income, and currently represents about 4% of all pharmaceutical market sales, totalling roughly $80 million (NHP Consulting, Saudi Arabia, 2016). In 2019, dietary supplement sales in Saudi Arabia are expected to rise to SR 875 million (Altamimi, 2019). This is attributable to the increased interest in the importance of maintaining one’s general health, wellbeing and protection from disease (Altamimi, 2019). According to a study conducted on the Northern border of Saudi Arabia, the use of vitamin supplements was reported to be 62% out of 400 participants (Abida et al., 2015). Another study in Saudi Arabia found the rate of dietary supplement utilization to be 59.4% among 736 adolescents (Musaiger and Abahussain, 2015). However, most of the studies carried out in Saudi have focused on one region or within a specific institution.

There are many reasons to consume nutritional supplements, including illness prevention defence against infection, disease, or health problems such as colds, stress, cardiovascular disease, osteoporosis symptoms, cancer, and dental caries. Supplements may also be used to increase energy levels, fight fatigue, boost physical performance, and correct several lifestyle deficiencies (Altamimi, 2019, Dwyer et al., 2013). Numerous micronutrients work as antioxidants, such as vitamin E, vitamin C, and beta-carotene (Muscogiuri et al., 2020). Antioxidants are substances that can increase interleukin-2 production, potentiate natural killer cell activity, increase the number of T-cell subsets, enhance lymphocyte response to mitogen, and increase the response to the influenza virus vaccine (Muscogiuri et al., 2020). Certain groups have higher nutritional demands, such as those who are pregnant or lactating, children and adolescents with unhealthy eating behaviours, the elderly, vegetarians, people with eating disorders and malabsorption, and people who eating fast or processed food (Alowais and Selim, 2019).

The effectiveness of vitamin supplements in protecting against COVID-19 infection has been suggested in research (Mattioli et al., 2020). For example, vitamin D has an important function as a modulator of immune properties, which include downregulation of pro-inflammatory cytokines (Greiller and Martineau, 2015, Martineau et al., 2017, Panarese et al., 2019). It is conceivable that the defensive effect of vitamin D against COVID-19 is the suppression of inflammatory cytokines’ response and a significant decrease in the severity of acute respiratory distress syndrome (ARDS) (Mattioli et al., 2020). There are few studies demonstrating the use of dietary supplements during COVID-19 pandemic in Saudi Arabia (Alfawaz et al., 2021, Hafiz et al., 2023). However study by Hafiz et al 2023, included only 500 participants in which most of them were from the middle region of Saudi Arabia (Hafiz et al., 2023). One the other hand, the study by Alfawaz et al,2021 did not measure consumption of certain supplements (Alfawaz et al., 2021).

This study, therefore, aims to measure the consumption of dietary supplements among the adult population in Saudi Arabia during the COVID-19 lockdown.

## 2 Materials and Methods

### 2.1 Study design and participants

This online cross-sectional survey was administered between 15 June 2020 and 15 August 2020. The number of confirmed cases of COVID-19 in Saudi Arabia rose from 41,014 to 98,869 throughout this period (Molfino et al., 2014). A lockdown means curfews for the sake of this research. Everyone age 18–65 who lives in Saudi Arabia, has permanent residency in the country, and has an internet connection was considered qualified to participate in the poll. We requested email addresses from everyone who took the survey, but just for that purpose and to ensure nobody answered the same question twice. None of the participants were required to use any type of supplement in order to participate.

Exclusion criteria was individuals with incomplete data, individuals who used unauthorized supplements were excluded or not meeting targeted age group.

A self-administered questionnaire was sent out to the participants using electronic means, such as Twitter and WhatsApp all over Saudi Arabia. Prior to the start of the trial, informed consent was sought from all participants.

The study was conducted in accordance with the Declaration of Helsinki, and approved by the Institutional Review Board (Biomedical Ethics Committee at Um Al-Qura University, Makkah City (HAPO-02-K-012–2020-10–447).

### 2.2 Questionnaire

The questionnaire included a cover letter in Arabic and English. It collected socio-demographic data and responses. Participants must respond to the same question about their routines before and after the lockdown. The questionnaire underwent several adjustments after being reviewed by experts in the field to increase its reliability and scientific value. Cronbach's was found to be outstanding (84%), indicating that the questionnaire's reliability and validity were confirmed in a pilot study (N = 75 participants). After the survey was finished, it was uploaded to a website and shared across several social media platforms all over the country. Each of the following topics was covered in the questionnaire:

(1) Age, sex, marital status, citizenship, income, number of dependents, education level, occupation, and area are all anthropometric and socio-demographic traits. Income levels were defined relative to the purchasing power of the Saudi Arabian Riyal (1 SAR = 0.266 USD). These factors were included to measure their influnce of supplments consumption.
(2) Supplemnts consumptionin general and a list of dietary supplements taken, such as vitamin D, selenium, zinc, and vitamin C and the reason of consumption. Then they were asked about the reason of using supplements and the amount of money they spend monthly. Last quastion was about if their consumptionhas changed during the pandemic.

### 2.3 Sample size calcuation and data analysis

In order to get a 95% confidence interval (CI) with a precision of 2%, a sample size of 1255 was needed to test the hypothesis that supplement use is as every day as stated in the literature (among Saudis, that is: 22%)(Alfawzan et al., 2022). A questionnaire was issued to 1572 prospective respondents to reduce the projected non-response rate of 20%.

The data were analyzed with SPSS 16.5. (SPSS Inc., Chicago, IL, USA). The results for the continuous variables were shown as means and standard deviations, while the results for the categorical variables were shown as numbers of occurrences (N) and percentages (%). To compare two categorical variables, the chi-square test was applied. Differentiating between normally distributed continuous variables was accomplished with the help of the independent *t*-test. Before and after the lockdown, dietary differences were analyzed using the chi-square test. In order to determine what factors are associated with DS consumption, with supplementation serving as the dependent variable and socio-demographic factors as the independent variables. A p-value below 0.05 was considered statistically significant.

## 3 Results

A total of 1572 participants from all parts of the country completed the study: 1276 (81.2%) female and 296 (18.8%) male. The demographic details of the participants are shown in Table 1. Around 1246 of the women (97.6%) were not pregnant, and 30 (2.4%) were pregnant. Furthermore, 43 (3.4%) were breastfeeding, whereas 1233 (97%) were not breastfeeding.

**Table 1:** PARTICIPANTS’ SOCIODEMOGRAPHIC CHARACTERISTICS.

**Demographic Characteristics**	**N (%)**
**Age**	
18–39	1082 (68.80%)
40–50	289 (18.40%)
51–64	182 (11.60%)
65 years and older	19 (1.20%)
**Nationality**	
Saudi	1463 (93.1%)
Non-Saudi	109 (6.9%)
**Place of Residence**	
Central Region	88 (5.60%)
Eastern Region	41 (2.60%)
Northern Region	14 (0.90%)
Western Region	1360 (86.50%)
Southern Region	69 (4.40%)
**Marital Status**	
Single	739 (47.00%)
Married	749 (47.60%)
Divorced/Widowed/Separated	84 (5.30%)
**Education**	
High school and lower education	344 (21.90%)
Diploma and Bachelor	998 (63.50%)
Higher education (Master/PhD)	230 (14.60%)
**Job**	
In the public sector	393 (25.00%)
In the health sector	108 (6.90%)
In the private sector	137 (8.70%)
Running their own business	20 (1.30%)
Currently not working	516 (32.80%)
Student	398 (25.30%)
**During the quarantine period, did your job require making contact with others?**	
Yes	322 (20.5%)
No	1250 (79.5%)
**Monthly Income**	
Less than 5000 SR/month	739 (47.00%)
5000–10000 SR/month	304 (19.30%)
10000–15000 SR/month	276 (17.60%)
15000–20000 SR/month	145 (9.20%)
More than 20,000 SR/month	108 (6.90%)
**Smoking**	
Smokers	145 (9.2%)
Non-smokers	1330 (84.6%)
Occasionally	97 (6.2%)
**BMI**	
Underweight	144 (9.20%)
Normal Weight	582 (37.00%)
Overweight	407 (25.90%)
Obesity class 1	263 (16.70%)
Obesity class 2	104 (6.60%)
Obesity class 3	72 (4.60%)

A total of 1330 (84.6%) participants were non-smokers. Regarding BMI, 582 (37%) participants were normal weight and 407 (25.90%) overweight. The mean number of family members in the household was 5 with ± 2.5 standard deviation. The minimum and maximum number of family members were 1 and 16, respectively.

The majority of the participants were healthy and did not consume any medication or have a history of surgery, as shown in Table 2.

**Table 2:** MEDICAL AND SURGICAL HISTORY (n = 1572).

**Question**	**n**	**%**
**Q1 – Are you currently using any medication?**		
No, I am not	1241 (78.9%)	
Yes, for Hypertension	107 (6.8%)	
Yes, for Endocrine Disease	95 (6%)	
Yes, for Diabetes Mellitus 2	74 (4.7%)	
Yes, for Bone Disease	70 (4.5%)	
Yes, for GIT disease	54 (3.4%)	
Yes, for Diabetes Mellitus 1	31 (2%)	
Yes, for Autoimmune Disease	30 (1.9%)	
Yes, for Heart Disease	19 (1.2%)	
Yes, for Kidney Disease	12 (0.8%)	
**Surgery History**		
None	1506 (95.8%)	
Sleeve gastrectomy	55 (3.5%)	
Colectomy (partial/total)	7 (0.4%)	
Dual partitioning process (gastric sleeve and path diversion)	6 (0.4%)	
Gastric bypass operation	2 (0.1%)	
Gastrectomy (partial/total)	2 (0.1%)	

**Fig. 1:**
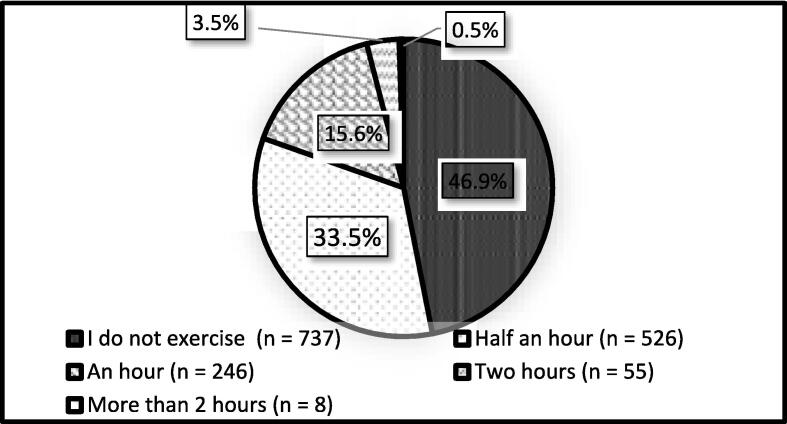
Physical activity levels of respondents.

Fig. 1 illustrates that 46.9% of the participants were sedentary and 737 participants did not exercise, whereas 526 (33.5%) participants exercised for half an hour, 246 (15.6%) exercised for an hour and 55 (3.5%) and 8 (0.5%) participants worked out for two hours and more than two hours, respectively.

### 3.1 Supplement consumption

As Table 3 demonstrates, 752 (47.84%) participants said that they had never heard of multivitamins, and 473 (30.09%) said that they had never consumed. In contrast, 197 (12.53%) participants reported taking multivitamins daily. Similarly, when asked about an antioxidant combination, 1428 (90.84%) said that they had neither heard of it nor consumed. When the participants were asked about fairly common vitamins and minerals such as vitamins A, C, D and E, a large proportion of them knew about these vitamins, though the majority of them had never supplemented with them. When asked about beta-carotene (A), B-complex, B12 (cyanocobalamin), calcium, chromium, folate, iron, magnesium, potassium, selenium, zinc, omega-3, cod liver oil, biotin and ginseng, approximately 70% of the participants knew about them, but most had never used.

**Table 3:** SUPPLEMENTS CONSUMED BY PARTICIPANTS (n = 1572).

**Supplement**	**Never heard of it**	**Never**	**Once/Month**	**Once/Week**	**2–6 times/week**	**Daily**
Multiple Vitamins/Minerals	752	473	41	53	56	197
	(47.83)	(30.08)	(2.6)	(3.37)	(3.56)	(12.53)
Antioxidant Combination	771	657	36	33	35	40
	(49.04)	(41.79)	(2.29)	(2.09)	(2.22)	(2.54)
Vitamin A	243	996	79	78	73	103
	(15.45)	(63.35)	(5.02)	(4.96)	(4.64)	(6.55)
Vitamin C	47	661	256	174	195	239
	(2.98)	(42.04)	(16.28)	(11.06)	(12.4)	(15.2)
Vitamin D	42	693	225	311	97	204
	(2.98)	(42.04)	(16.28)	(11.06)	(12.4)	(15.2)
Vitamin E	167	1053	90	84	73	105
	(2.98)	(42.04)	(16.28)	(11.06)	(12.4)	(15.2)
Beta-carotene (A)	425	963	46	41	37	60
	(27.03)	(61.25)	(2.92)	(2.6)	(2.35)	(3.81)
B-Complex	233	930	88	82	69	170
	(14.82)	(59.16)	(5.59)	(5.21)	(4.38)	(10.81)
B12 (Cyanocobalamin) alone	197	917	115	84	76	183
	(12.53)	(58.33)	(7.31)	(5.34)	(4.83)	(11.64)
Calcium	51	875	113	110	123	300
	(3.24)	(55.66)	(7.18)	(6.99)	(7.82)	(19.08)
Chromium	559	911	27	20	18	37
	(35.55)	(57.95)	(1.71)	(1.27)	(1.14)	(2.35)
Folate (Folic acid, Folacin)	163	1034	108	60	55	152
	(10.36)	(65.77)	(6.87)	(3.81)	(3.49)	(9.66)
Iron	55	889	129	106	109	284
	(3.49)	(56.55)	(8.2)	(6.74)	(6.93)	(18.06)
Magnesium	131	1105	72	73	70	121
	(8.33)	(70.29)	(4.58)	(4.64)	(4.45)	(7.69)
Potassium	132	1126	82	74	66	92
	(8.39)	(71.62)	(5.21)	(4.7)	(4.19)	(5.85)
Selenium	400	1038	28	31	34	41
	(25.44)	(66.03)	(1.78)	(1.97)	(2.16)	(2.6)
Zinc	141	1064	96	73	70	128
	(8.96)	(67.68)	(6.1)	(4.64)	(4.45)	(8.14)
Omega-3	134	951	125	104	81	177
	(8.52)	(60.49)	(7.95)	(6.61)	(5.15)	(11.25)
Cod liver oil	151	1139	89	49	45	99
	(9.6)	(72.45)	(5.66)	(3.11)	(2.86)	(6.29)
Biotin	375	1021	35	26	38	77
	(23.85)	(64.94)	(2.22)	(1.65)	(2.41)	(4.89)
Collagen	190	1139	79	54	44	66
	(12.08)	(72.45)	(5.02)	(3.43)	(2.79)	(4.19)
Ginseng	341	1090	46	28	30	37
	(21.69)	(69.33)	(2.92)	(1.78)	(1.9)	(2.35)

### 3.2 Beliefs and attitudes toward supplements

**Fig. 2:**
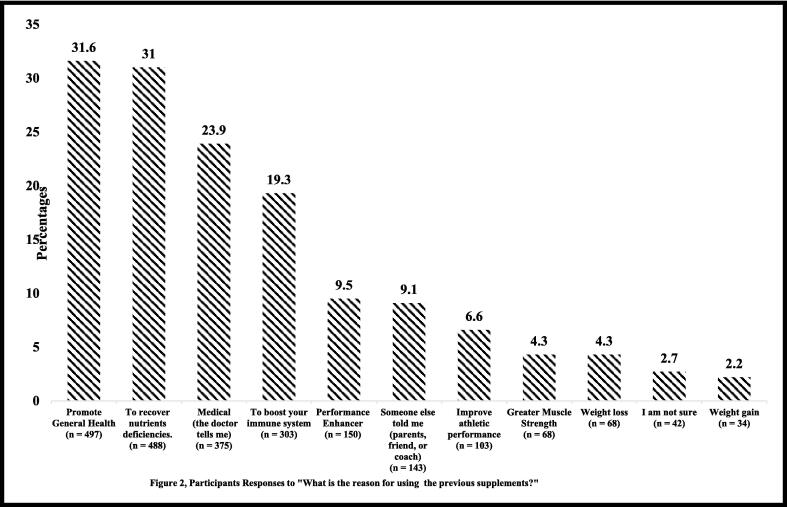
Reason for using supplements.

The motivations behind consuming supplements varied between individuals. The number of individuals who consumed supplements to promote general health and to correct nutrient deficiencies was 497 (31.6%) and 488 (31%), respectively. A total of 375 (23.9%) participants consumed supplements on the recommendation of a doctor, while 303 (19.3%) participants wanted to boost their immune system. <10% of individuals consumed the supplements to enhance their physical performance or on the recommendation of their friends and family (Fig. 2).

The monthly cost of supplements was different between individuals. No money was spent by 841 (53.5%) of the participants because they did not consume supplements. While 425 (27%) of the participants only spent 50 to 100 SAR, 202 (12.8%) spent 100 to 500 SAR, 29 (1.8%) spent 500 to 1000 SAR, and 67 (4.3%) participants were given their supplements at no cost from the hospital.

**Fig. 3:**
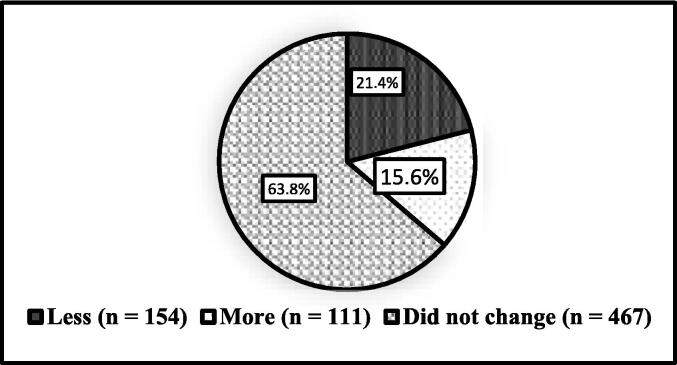
Change in supplement consumption pattern due to quarantine.

Fig. 3 represents participants response for the question about the change in supplement consumption during the COVID-19 pandemic. In total, 467 (63.8%) of the participants did not change their consumption patterns, while 111 (15.6%) consumed more supplements and 154 (21.4%) consumed fewer supplements during the pandemic.

### 3.3 Factors influencing supplement intake during the pandemic

Table 4 illustrates the association between sociodemographic factors and the overall consumption of supplements. A significant association was found between gender and consumption of supplements (*P* < 0.002), whereas a higher rate of supplement consumption was seen in females versus males (81% and 73%, respectively). A significant association was also found between age and consumption of supplements (*P* < 0.002): The older the age group, the higher the rate of supplement consumption. Marital status was also observed to be significantly associated with consumption of supplements (*P* < 0.001), where the highest rate of consumption was seen in married people. Moreover, education was significantly correlated with the consumption of supplements (*P* = 0.02), where the higher the education, the higher the rate of supplement consumption. Job was also significantly related to the consumption of supplements (*P* = 0.002), as was monthly income (*P* = 0.006), where those with the lowest income had the lowest rate of consumption, while those with the highest income had the highest rate of consumption. Pregnancy was also significantly correlated with the consumption of supplements (*P* < 0.007), where a higher rate of consumption was observed among pregnant women.

**Table 4:** SOCIODEMOGRAPHIC FACTORS ASSOCIATED WITH CONSUMPTION OF SUPPLEMENTS.

**Demographic Characteristics**	**Currently consume any supplements?**		**P-value**
	**No**	**Yes**	
**Gender**			0.002*
Male	80 (27%)	216 (73%)	
Female	242 (19%)	1034 (81%)	
**Age**			0.002*
18–39	249 (23%)	833 (77%)	
40–50	46 (15.9%)	243 (84.1%)	
51–64	26 (14.3%)	156 (85.7%)	
65 years and older	1 (5.3%)	18 (94.7%)	
**Nationality**			0.511
Saudi	297 (20.3%)	1166 (79.7%)	
Non-Saudi	25 (22.9%)	84 (77.1%)	
**Place of Residence**			0.186
Central Region	22 (25%)	66 (75%)	
Eastern Region	9 (22%)	32 (78%)	
Northern Region	2 (14.3%)	12 (85.7%)	
Western Region	268 (19.7%)	1092 (80.3%)	
Southern Region	21 (30.4%)	48 (69.6%)	
**Marital Status**			< 0.001**
Single	186 (25.2%)	553 (74.8%)	
Married	120 (16%)	629 (84%)	
Divorced/Widowed/Separated	16 (19%)	68 (81%)	
**Education**			0.02*
High school and lower education	87 (25.3%)	257 (74.7%)	
Diploma and Bachelor	198 (19.8%)	800 (80.2%)	
Higher education (Master/PhD)	37 (16.1%)	193 (83.9%)	
**Job**			0.002*
In the public sector	70 (17.8%)	232 (82.2%)	
In the health sector	20 (18.5%)	88 (81.5%)	
In the private sector	32 (23.4%)	105 (76.6%)	
Running their own business	0 (0%)	20 (100%)	
Currently not working	94 (18.2%)	422 (81.8%)	
Student	106 (26.6%)	292 (73.4%)	
**During the quarantine period, did your job require making contact with others?**			0.443
Yes	61 (18.9%)	261 (81.1%)	
No	261 (20.9%)	989 (79.1%)	
**Monthly Income**			0.006*
Less than 5000 SR/month	181 (24.5%)	558 (75.5%)	
5000–10000 SR/month	52 (17.1%)	252 (82.9%)	
10000–15000 SR/month	49 (17.8%)	227 (82.2%)	
15000–20000 SR/month	25 (17.2%)	120 (82.8%)	
More than 20,000 SR/month	15 (13.9%)	93 (86.1%)	
**Smoking**			0.402
Yes	28 (19.3%)	117 (80.7%)	
No	279 (21%)	1051 (79%)	
Occasionally	15 (15.5%)	82 (84.5%)	
**BMI**			0.097
Underweight	38 (26.4%)	106 (73.6%)	
Normal Weight	125 (21.5%)	457 (78.5%)	
Overweight	80 (19.7%)	327 (80.3%	
Obesity class 1	41 (15.6%)	222 (84.4%)	
Obesity class 2	19 (18.3%)	85 (81.7%)	
Obesity class 3	19 (26.4%)	53 (73.6%)	
**Are you currently pregnant?**			0.007*
Yes	0 (0%)	30 (100%)	
No	242 (19.4%)	1004 (80.6%)	
Are you currently breastfeeding?			0.100
Yes	4 (9.3%)	39 (90.7%)	
No	238 (19.3%)	995 (80.7%)	
**If you exercise, how much time do you usually spend daily?**			0.097
I do not exercise	172 (23.3%)	565 (76.7%)	
Half an hour	95 (18.1%)	431 (81.9%)	
An hour	44 (17.9%)	202 (82.1%)	
Two hours	10 (18.2%)	45 (81.8%)	
More than two hours	1 (12.5%)	7 (87.5%)	

* Significance level P ≤ 0.05.

** Significance level P ≤ 0.005.

Table 5 illustrates the factors associated with the consumption of vitamin C and D. Pregnancy was significantly associated with the consumption of vitamin C (P = 0.004), where a higher rate of vitamin C consumption was seen in non-pregnant women. Age was significantly associated with vitamin D consumption (*P* < 0.001): The older the age, the higher the consumption. Education was also significantly correlated with vitamin D consumption (*P* = 0.018), where the higher the education level, the higher the rate of vitamin D consumption. Moreover, monthly income demonstrated a significant relationship with vitamin D consumption (*P* = 0.035).

**Table 5:** FACTORS ASSOCIATED WITH CONSUMPTION OF VITAMIN C AND D.

**Demographic Characteristics**	**Vitamin C**		**P-value**	**Vitamin D**		**P-value**
	**No**	**Yes**		**No**	**Yes**	
**Age**			00.87			
18–39	502 (46.4%)	580 (53.6%)		544 (50.3%)	538 (49.7%)	<0.001**
40–50	132 (45.7%)	157 (54.3%)		129 (44.6%)	160 (55.4%)	
51–64	68 (37.4%)	114 (62.6%)		59 (32.4%)	123 (67.6%)	
65 years and older	6 (31.6%)	13 (68.4%)		3 (15.8%)	16 (84.2%)	
**Place of Residence**			0.683			
Central Region	41 (46.6%)	47 (53.4%)		41 (46.6%)	47 (53.4%)	0.412
Eastern Region	16 (39%)	25 (61%)		21 (51.2%)	20 (48.8%)	
Northern Region	4 (28.6%)	10 (71.4%)		3 (21.4%)	11 (78.6%)	
Western Region	615 (45.2%)	745 (54.8%)		683 (46.9%)	722 (53.1%)	
Southern Region	32 (46.4%)	37 (53.6%)		32 (46.4%)	37 (53.6%)	
**Education**			0.893			
High school and lower education	158 (45.9%)	186 (54.1%)		178 (51.2%)	168 (48.8%)	0.018*
Diploma and Bachelor	445 (44.6%)	553 (55.4%)		469 (47%)	529 (53%)	
Higher education (Master/PhD)	105 (45.7%)	125 (54.3%)		90 (39.1%)	140 (60.9%)	
**During the quarantine period, did your job require making contact with others?**			0.708			
Yes	148 (46%)	174 (54%)		146 (45.3%)	176 (54.7%)	0.568
No	560 (44.8%)	690 (55.2%)		589 (47.1%)	661 (52.9%)	
**Monthly Income**			0.367			
Less than 5000 SR/month	334 (45.2%)	405 (54.8%)		373 (50.5%)	366 (49.5%)	0.035*
5000–10000 SR/month	131 (43.1%)	173 (56.9%)		135 (44.4%)	169 (55.6%)	
10000–15000 SR/month	127 (46%)	149 (54%)		127 (46%)	149 (54%)	
15000–20000 SR/month	59 (40.7%)	86 (59.3%)		59 (40.7%)	86 (59.3%)	
More than 20,000 SR/month	57 (52.8%)	51 (47.2%)		41 (38%)	67 (62%)	
**Are you currently pregnant?**			0.004*			
Yes	21 (70%)	9 (30%)		15 (50%)	15 (50%)	0.576
No	543 (43.6%)	703 (56.4%)		559 (44.9%)	687 (55.1%)	

* Significance level 0.05.

** Significance level 0.005.

Table 6 details the factors associated with the consumption of calcium and iron. Age was significantly associated with calcium consumption (*P* = 0.008), where the higher age groups had higher rates of calcium consumption. Pregnancy was also significantly associated with calcium consumption (*P* < 0.001). There was no significant association between consumption and place of residence, education and income. Participants in the age group 18–39 consumed a higher amount of iron supplements than any other age group. People of the southern and western regions consumed more iron than other parts of the country. Participants who were in high school and earned less money spent more on iron supplements than those who were more educated or were earning more. Notably, 90% of the women who were pregnant were consuming iron supplements.

**Table 6:** FACTORS ASSOCIATED WITH CONSUMPTION OF IRON AND CALCIUM.

**Demographic Characteristics**	**Iron**		**P-value**	**Calcium**		**P-value**
	**No**	**Yes**		**Yes**	**No**	
**Age**			0.230			
18–39	634 (58.6%)	448 (41.4%)		771 (71.3%)	311 (28.7%)	0.005*
40–50	177 (61.2%)	112 (38.8%)		191 (66.1%)	98 (33.9%)	
51–64	120 (65.9%)	62 (34.1%)		115 (63.2%)	67 (36.8%)	
65 years and older	13 (68.4%)	6 (31.6%)		8 (42.1%)	11 (57.9%)	
**Place of Residence**			0.603			
Central Region	60 (68.2%)	28 (31.8%)		66 (75%)	22 (25%)	0.366
Eastern Region	25 (61%)	16 (39%)		28 (68.3%)	13 (31.7%)	
Northern Region	9 (64.3%)	5 (35.7%)		7 (50%)	7 (50%)	
Western Region	809 (59.5%)	551 (40.5%)		934 (68.7%)	426 (31.3%)	
Southern Region	41 (59.4%)	28 (40.6%)		50 (72.5%)	19 (27.5%)	
**Education**			0.850			
High school and lower education	194 (56.4%)	150 (43.6%)		249 (72.4%)	95 (27.6%)	0.077
Diploma and Bachelor	599 (60%)	399 (40%)		690 (69.1%)	308 (30.9%)	
Higher education (Master/PhD)	151 (65.7%)	79 (34.3%)		146 (63.5%)	84 (36.5%)	
**During the quarantine period, did your job require making contact with others?**			0.017*	146 (63.5%)	84 (36.5%)	0.710
Yes	212 (65.8%)	110 (34.2%)		225 (69.9%)	97 (30.1%)	
No	732 (58.6%)	518 (41.4%)		860 (68.8%)	390 (31.2%)	
**Monthly Income**			0.033*			0.150
Less than 5000 SR/month	426 (57.6%)	313 (42.4%)		522 (70.6%)	217 (29.4%)	
5000–10000 SR/month	177 (58.2%)	127 (41.8%)		210 (69.1%)	94 (30.9%)	
10000–15000 SR/month	167 (60.5%)	109 (39.5%)		189 (68.5%)	87 (31.5%)	
15000–20000 SR/month	98 (67.6%)	47 (32.4%)		101 (69.7%)	44 (30.3%)	
More than 20,000 SR/month	76 (70.4%)	32 (29.6%)		63 (58.3%)	45 (41.7%)	
**Are you currently pregnant?**			<0.001^**^			0.776
Yes	3 (10%)	27 (90%)		20 (66.7%)	10 (33.3%)	
No	725 (58.2%)	521 (41.8%)		861 (69.1%)	385 (30.9%)	

## 4 Discussion

The main purpose of the current research was to study the consumption of dietary supplements in Saudi Arabia during the COVID-19 pandemic lockdown. The findings revealed substantial information regarding the supplement consumption habits of the Saudi general public.

### 4.1 Supplement consumption during COVID-19 pandemic

The results of this study did not show any significant increase in supplement consumption patterns during the pandemic; in fact, some participants reduced their consumption of supplements during quarantine. Possible reasons include the inability to leave home due to curfews, fear of getting infected by the virus, economic burden and dependence on natural sources for supplements. Vitamin D, vitamin E, iron, calcium, and omega-3 were those most consumed by the participants. The high consumption of vitamins D and E could be due to the extensive reporting of research demonstrating their role in aiding recovery from COVID-19, in addition to their antioxidant and anti-inflammatory properties. Vitamin D supplementation has been of particular interest, and its use is being popularized, as low vitamin D levels have been associated with an elevated risk of ARDS and infectious diseases, including upper respiratory tract infections. In addition, it is now the target of many prophylactic and therapeutic clinical trials. Overall, poor vitamin D status seems to be associated with an increased risk of COVID-19: It has been found that severely ill COVID-19 patients appear to be deficient or have suboptimal levels of serum 25-hydroxyvitamin D (Lordan, 2021). Additionally, vitamin C was reported to be consumed regularly this has been found in Saudi Arabia that vitamin C was taken during the COVID-19 pandemic by almost all dietary supplement users (Hamulka et al., 2021). Moreover, according to a cross-sectional study conducted to examine the impact of COVID-19 quarantine on dietary habits and physical activity in Saudi Arabia, vitamin C was the most consumed dietary supplement during quarantine, followed by vitamin D and multivitamins, respectively (Bakhsh et al., 2021). It is noteworthy that the use of dietary supplements was significantly associated with the participants’ education level, which is consistent with findings from several previous studies (Alyami et al., 2020, Bakhsh et al., 2021).

Regarding omega-3 high consumption in this study. This might be due to evidence suggesting the beneficial effect of omeg-3 consumption such as reduction in the primary end point for a combination of death, nonfatal myocardial infarction, and nonfatal stroke (Manor et al., 2012, NCT04386525, 2020, Patel et al., 2009).

### 4.2 Beliefs and attitudes toward supplements

The largest proportion of the participants were using supplements to promote general health. This has been found earlier (Ishihara et al., 2003, Kaufman et al., 2002). It has been observed that supplement users as having somewhat positive attitude toward their health(Ishihara et al., 2003). They were more likely to be affluent, health conscious, less likely to smoke, and likely to eat more healthy food (Chakrabarti et al., 2020, Shah and Barritt, 2022). Doctors’ advice was a popular reason to consume supplements. This has been observed previously.

### 4.3 Factors influencing dietary supplements consumption

The notable difference in dietary supplement intake between pregnant and non-pregnant women can be attributed to the fact that certain micronutrients are vital for improving pregnancy outcomes (Mousa et al., 2019). Folic acid, iron, zinc, calcium, vitamin C and vitamin D are the main micronutrients for which one’s requirement physiologically increases during pregnancy and lactation (Marangoni et al., 2016). Iron supplements, in particular, have been routinely recommended in pregnancy because iron needs nearly double during this time (Kominiarek and Rajan, 2016). Demand for iron increases from 0.8 to up to 7.5 mg/day of absorbed ferritin; this increased demand is needed to expand maternal erythrocyte mass, fulfil foetal iron needs and compensate for iron losses (e.g. blood loss at delivery). Maternal iron requirements, therefore, exceed average absorbable iron intakes; in turn, the risk of developing iron-deficiency anaemia is increased in pregnancy (Mousa et al., 2019).

The high consumption of calcium in pregnancy is attributed to the fact that calcium is actively transported across the placenta and maternal calcium demands increase, particularly during the third trimester. Increased calcium needs may therefore be met by diet alone (1.2 g/day recommended); however, supplementation of 0.3–2.0 g/day is recommended by some to preserve maternal calcium balance and bone density and to support foetal development, particularly in women with low dietary calcium intake (Mousa et al., 2019).

Age was a factor that significantly influenced dietary supplement consumption in this study. Studies on the demographic characteristics of dietary supplements users also found that they were mainly females, with a higher education and of young age (20–39 years) (Braun and Venter, 2008, Ishihara et al., 2003). In the current study, young participants between the ages of 18 and 39 consumed more iron supplements. Many factors might increase the need to iron such as pregnancy, childhood, heavy exercise and some medical conditions. Older participants who were above the age of 65 consumed more omega-3 supplements, as they may have various effects on cognitive health due to their anti- or pro-inflammatory properties; for example, regular consumption of fatty fish has been associated with slower cognitive decline. Additionally, previous longitudinal studies have reported relationships between higher blood concentrations of polyunsaturated fatty acids (PUFAs), including omega-3, and decreased risk of dementia (van Lent et al., 2021). Omega-3 supplements can modulate inflammation, hyperlipidemia, platelet aggregation and hypertension, which explains why those 65 and older consumed more omega-3 supplements than other age groups (Molfino et al., 2014). The participants consumed these particular minerals the most, as they might have known about the benefits or were following medical advice.

### 4.4 Strength and limitation

The strength of this study is that the participants were of various ages, genders, nationalities, areas of residency, marital status, fields of education, fields of work and organizations, and they varied in physical condition and health. Additionally we focused on the most consumed supplements.

Because this study is cross-sectional, we cannot draw firm conclusions about cause and effect. In addition, we are unable to compare our results to those of other studies since so few have examined the use of DS according to gender and COVID-19 status. Our findings highlight the small sample size of dietary consumption factors and the likelihood that we have overlooked some crucial aspects of DS use. An online survey runs the risk of missing some potential respondents due to factors like distance or lack of access to the internet.

Including pregnat women in the study migh concoder as a limitation as women in these stages of life tend to be more cautious, which can influence the results. Moreover, omiting to measure the consumption of other supplments and only including vitamins, miniral and (omega-3, collagen, and ginseng) is one of the limitations.

Despite these restrictions, the present study provides proof and guidance for future studies. It suggests that health organizations prioritize spreading nutrition knowledge about healthy nutrition habits and quality food preferences as sustainable dietary options, especially in the context of existing pandemic contexts. When it comes to DS, consumers need to be educated effectively about credible resources where they can find accurate information. They also need to be made aware of and comprehend the potential risks and benefits associated with using DS to treat and prevent COVID-19. More research and clinical studies are needed to identify the significance of DS in preventing COVID-19 infection and evaluate whether DSs demonstrate any true therapeutic efficacy against this pandemic disease.

## 5 Conclusions

Summarizing the research results, it can be concluded that a substantial portion of Saudi Arabia’s population consumes supplements, the most common of which being iron, calcium, omega-3 and vitamin D. Our results showed that consumption of DS was not influnced by COVID-19 pandemic. Women supplement takers outnumbered men by a wide margin. In addition to age, finincial status, and education level, were substantial factor of DS consumption. Furthermore, for patient safety, DS must be supported by reliable data. The attitudes and goals of people who take dietary supplements for their health can be documented in future research.

## Institutional Review Board Statement

The study was conducted in accordance with the Declaration of Helsinki, and approved by the Institutional Review Board (Biomedical Ethics Committee at Um Al-Qura University, Makkah City (HAPO-02-K-012–2020-10–447).

## Informed Consent Statement

“Informed consent was obtained from all subjects involved in the study.”

## CRediT authorship contribution statement

**Wedad Azhar:** Validation, Writing – review & editing. **Kholod Al-Otaibi:** Methodology, Writing – original draft. **Wafaa F. Abusudah:** Investigation. **Firas Azzeh:** Conceptualization, Writing – review & editing. **Alaa Qhadi:** Validation, Writing – review & editing. **Walaa E. Alhassani:** Formal analysis, Visualization, Supervision. **Najlaa H. Almohmadi:** Formal analysis, Supervision. **Taqwa Bushnaq:** Validation, Project administration. **Bayan Tashkandi:** Project administration. **Nouf Abdullah Alharbi:** Investigation, Visualization. **Abrar Babteen:** Writing – review & editing, Project administration. **Mai Ghabashi:** Writing – original draft. **Yara Kamfar:** Methodology, Supervision. **Khloud Ghafouri:** Conceptualization, Methodology.

## Declaration of Competing Interest

The authors declare that they have no known competing financial interests or personal relationships that could have appeared to influence the work reported in this paper.
